# Alpha-2 nicotinic acetylcholine receptors regulate spectral integration in auditory cortex

**DOI:** 10.3389/fncir.2024.1492452

**Published:** 2024-11-01

**Authors:** Irakli Intskirveli, Susan Gil, Ronit Lazar, Raju Metherate

**Affiliations:** Department of Neurobiology and Behavior, Center for Hearing Research, University of California, Irvine, Irvine, CA, United States

**Keywords:** nicotine, mouse, receptive field, electrophysiology, current-source density, neuromodulation, martinotti

## Abstract

**Introduction:**

In primary auditory cortex (A1), nicotinic acetylcholine receptors (nAChRs) containing α2 subunits are expressed in layer 5 Martinotti cells (MCs)—inhibitory interneurons that send a main axon to superficial layers to inhibit distal apical dendrites of pyramidal cells (PCs). MCs also contact interneurons in supragranular layers that, in turn, inhibit PCs. Thus, MCs may regulate PCs via inhibition and disinhibition, respectively, of distal and proximal apical dendrites. Auditory inputs to PCs include thalamocortical inputs to middle layers relaying information about characteristic frequency (CF) and near-CF stimuli, and intracortical long-distance (“horizontal”) projections to multiple layers carrying information about spectrally distant (“nonCF”) stimuli. CF and nonCF inputs integrate to create broad frequency receptive fields (RFs). Systemic administration of nicotine activates nAChRs to “sharpen” RFs—to increase gain within a narrowed RF—resulting in enhanced responses to CF stimuli and reduced responses to nonCF stimuli. While nicotinic mechanisms to increase gain have been identified, the mechanism underlying RF narrowing is unknown.

**Methods:**

Here, we examine the role of α2 nAChRs in mice with α2 nAChR-expressing neurons labeled fluorescently, and in mice with α2 nAChRs genetically deleted.

**Results:**

The distribution of fluorescent neurons in auditory cortex was consistent with previous studies demonstrating α2 nAChRs in layer 5 MCs, including nonpyramidal somata in layer 5 and dense processes in layer 1. We also observed label in subcortical auditory regions, including processes, but no somata, in the medial geniculate body, and both fibers and somata in the inferior colliculus. Using electrophysiological (current-source density) recordings in α2 nAChR knock-out mice, we found that systemic nicotine failed to enhance CF-evoked inputs to layer 4, suggesting a role for subcortical α2 nAChRs, and failed to reduce nonCF-evoked responses, suggesting that α2 nAChRs regulate horizontal projections to produce RF narrowing.

**Discussion:**

The results support the hypothesis that α2 nAChRs function to simultaneously enhance RF gain and narrow RF breadth in A1. Notably, a similar neural circuit may recur throughout cortex and hippocampus, suggesting widespread conserved functions regulated by α2 nAChRs.

## Introduction

Heteromeric nAChRs containing α4 and β2 subunits are by far the most common in the rodent forebrain, accounting for ~90% of nAChRs, while homomeric α7 nAChRs account for much of the remainder (Wada et al., 1989; Gotti et al., 2006; Mao et al., 2008). However, recent studies have shown that nAChRs containing α2 subunits, while relatively scarce (<3% in cortex) (Wada et al., 1989; Mao et al., 2008), may nonetheless play a key role in regulating cortical function. Notably, in primate cortex α2 nAChRs may be as common as α4 nAChRs, suggesting evolutionary pressure to favor functions mediated by α2 nAChRs (Han et al., 2000; Aridon et al., 2006).

Studies of α2 nAChRs in hippocampus have focused on oriens lacunosum-moleculare (OLM) cells in the CA1 region (Ishii et al., 2005; Nakauchi et al., 2007; Jia et al., 2009; Leao et al., 2012; Lotfipour et al., 2017; Hilscher et al., 2023). These somatostatin (SOM)-expressing interneurons have cell bodies in stratum oriens (SO), adjacent to PC somata, and send axons to stratum lacunosum moleculare (SLM) where they contact the distal apical dendrites of PCs to inhibit long distance inputs from entorhinal cortex (Leao et al., 2012; Hilscher et al., 2023). OLM cells also project to interneurons in the stratum radiatum (SR) that, in turn, inhibit PC proximal dendrites receiving Schaeffer collateral inputs from CA3 (Leao et al., 2012; Hilscher et al., 2023). Thus, OLM cells regulate information processing in PCs via simultaneous inhibition of distal apical dendrites and disinhibition of proximal apical dendrites. Uniquely among hippocampal neurons, OLM cells express α2 nAChRs (Ishii et al., 2005) that, unlike most nAChRs, exhibit little desensitization in response to prolonged exposure to nicotine (Jia et al., 2009). As a result, α2 nAChRs exert disproportional effects, relative to their scarcity, on neural circuits, systems-level processing, and behavior, in response to nicotine or endogenous acetylcholine (ACh) (Lotfipour et al., 2017; Siwani et al., 2018; Hilscher et al., 2023).

A similar neural circuit is found in cortex where α2 nAChRs are expressed in layer 5 MCs, interneurons that send prominent projections to layer 1 to inhibit distal apical dendrites of PCs and lesser projections to layers 2–5 where they contact other interneurons that, in turn, inhibit PCs (Wang et al., 2004; Hilscher et al., 2017; Nigro et al., 2018; Obermayer et al., 2018; Donato et al., 2023). Many MCs also express SOM (Hilscher et al., 2017). Layer 5 MCs, therefore, also mediate inhibition and disinhibition, respectively, of PC distal and proximal apical dendrites. In A1, potential targets of MCs include thalamocortical inputs to middle layers and intracortical “horizontal” inputs to middle and upper layers from distant regions within A1 (Kaur et al., 2004; Happel et al., 2010; Kratz and Manis, 2015; Intskirveli et al., 2016). Thus, layer 5 MCs resemble hippocampal OLM cells in terms of morphology, synaptic connectivity, and expression of α2 nAChRs (Hilscher et al., 2023). However, the functional role of MCs or α2 nAChRs in auditory processing is not known.

Thalamocortical inputs to A1 carry auditory information about CF and near-CF stimuli, whereas horizontal inputs carry information about spectrally distant “nonCF” stimuli and contribute to spectral integration by increasing the breadth of RFs (Kaur et al., 2004; Happel et al., 2010; Kratz and Manis, 2015; Intskirveli et al., 2016). In mouse A1, systemic nicotine produces a narrowing of RFs and, simultaneously, increased gain within the narrowed RF (Liang et al., 2008; Kawai et al., 2011; Intskirveli and Metherate, 2012; Askew et al., 2017). This RF “sharpening” has been demonstrated using two kinds of acoustic stimuli, either “notched noise” stimuli with varying notch widths to determine RF width and gain (Askew et al., 2017) or, more simply, using tones to demonstrate increased response to CF stimuli and decreased response to nonCF stimuli (~2 octaves from CF) (Kawai et al., 2011; Intskirveli and Metherate, 2012). Nicotinic mechanisms underlying enhanced CF-evoked responses include activation of heteromeric nAChRs to increase the excitability of thalamocortical axons and enhance inputs to A1 (Kawai et al., 2007; Intskirveli and Metherate, 2012; Askew et al., 2017). However, to date, no mechanism has been identified for nicotinic reduction of nonCF-evoked responses, but a plausible mechanism involves suppression of horizontal projections mediating nonCF inputs. Activation of α2 nAChRs-expressing MCs, therefore, is a potential mechanism for inhibiting horizontal projections to PCs. To address this issue, we examined the location and function of α2 nAChRs using mice with α2 nAChR-expressing neurons labeled fluorescently, and mice with α2 nAChRs genetically deleted. The results support the hypothesis that α2 nAChRs mediate RF narrowing (reduced responses to nonCF stimuli) and contribute, along with non-α2 nAChRs, to increased gain within the narrowed RF (increased responses to CF stimuli).

## Materials and methods

### Animals

For visualization of enhanced green fluorescent protein (EGFP) in cells expressing α2 nAChRs we used a transgenic FVB mouse line (Chrna2-EGFP) obtained cryopreserved from MMRRC (RRID: MMRRC 036130-UCD) and reanimated by the UC Irvine Transgenic Mouse Facility. Male and female mice were used for anatomy (60–90 days old) and *in vitro* electrophysiology (45–55 days old). For *in vivo* physiology, we used transgenic C57Bl/6 J mice, either α2 nAChRs knock-out (KO) mice (Chrna2^−/−^) or wild-type (WT) littermates from heterozygous breeders in a line obtained from Dr. Shahrdad Lotfipour, UC Irvine (Lotfipour et al., 2013) (RRID: MMRRC 030508-ICD). Auditory studies were conducted on young adults (60–90 days) with verified normal auditory thresholds, and with males and females included in each group in approximately equal numbers. All procedures were approved by the University of California, Irvine, Institutional Animal Care and Use Committee (IACUC).

### Anatomy

Animals were deeply anesthetized with urethane (0.7 g/kg, i.p.; Sigma) and xylazine (13 mg/kg; Akorn) and perfused transcardially with ice-cold phosphate buffered saline (PBS), followed by 4% paraformaldehyde in PBS. Brains were removed and placed in the same fixative. After overnight fixation the brain was rinsed in PBS and sectioned along the thalamocortical (Cruikshank et al., 2002) or coronal plane in 50 μm sections using a microtome (VT1000P, Leica Biosystems). Representative sections were selected and mounted on slides with Glycergel mounting medium (Dako). To capture images, we used a microscope (Zeiss Axioskop A1) equipped with a digital camera (Axiocam HRc) and fluorescent light source (X-Cite; 120 Q Series, EXFO Photonic Solution). Imaging software (Zeiss Zen 2.3) was used to store images.

### *In vitro* electrophysiology

Mice were anesthetized with isoflurane and decapitated. Brains were quickly removed into cold ACSF containing (in mM): 125 NaCl, 2.5 KCl, 25 NaHCO3, 1.25 KH2PO4, 1.2 MgSO4, 2.0 CaCl2, and 10 dextrose, bubbled with 95% O2/5% CO2. Auditory thalamocortical slices ~300 μm thick were prepared using a vibrating microtome (Leica VT1000s) as described (Cruikshank et al., 2002) with sections cut approximately 20 degrees off horizontal. Slices were placed in a holding chamber containing oxygenated ACSF at room temperature for 60 min before recording. For recording, a thalamocortical slice was transferred to a submersion chamber and maintained in continuous flow of ACSF (2.5–3 mL/min) at room temperature. Neurons were visualized using infrared differential interference contrast (Zeiss Axioskop 2), and cells with EGFP identified using a fluorescent light source (X-Cite; 120 Q Series). Whole-cell recordings were obtained with patch pipettes (2–6 MΩ) filled with (in mM): 135 K-gluconate, 1 KCl, 2 MgCl2, 1 Na-ATP, 0.5 Na-GTP, 1 EGTA, 10 HEPES (pH 7.3, 270 mOsm). Responses were acquired with a MultiClamp 700B amplifier (Molecular Devices) in current-clamp mode and AxoGraph software. Signals were amplified, low pass filtered at 2 kHz, and digitally sampled at 10 kHz. Series resistance (6–15 MΩ) was continuously monitored and data discarded if resistance changed more than 30%. The recording location in auditory cortex was based on previous studies (Cruikshank et al., 2002) and confirmed in some slices by a short-latency response to stimulation of the thalamocortical pathway. Nicotine hydrogen tartrate (Sigma) dissolved in saline was added to ACSF and bath applied (1–100 μM, free base).

### Acoustic physiology

Mice were anesthetized with urethane (0.7 g/kg, i.p.) and xylazine (13 mg/kg), supplemented as needed (urethane, 0.14 g/kg; xylazine, 1.3 mg/kg). Urethane was used due to its limited suppression of nAChR function (Hara and Harris, 2002; Tassonyi et al., 2002). Mice were placed in a sound-attenuating chamber (AC-3; IAC Acoustics) and maintained at 37°C on a heating pad. After securing the head in a stereotaxic frame (model 923; Kopf Instruments) using blunt ear bars, a midline incision was made and the skull cleared and secured using a custom head holder. A craniotomy was performed over the right auditory cortex and the exposed brain kept moist with warm saline. Ear bars were removed before presentation of acoustic stimuli.

A1 was identified by mapping with a glass micropipette (1 M NaCl, ~1 MΩ at 1 kHz) to record local field potentials (LFPs) evoked in response to a standard stimulus set (1–40 kHz in 2 kHz steps; 5–70 dB SPL in 5 dB steps). Acoustic stimuli were digitally synthesized and controlled (RP2.1 Enhanced Real-Time Processor; Tucker-Davis Technologies) and delivered from a speaker (FF-1 with SA-1 Stereo Power Amp; TDT) placed 3 cm from the left ear. Tones were 100 ms duration with 5 ms linear rise and fall ramps. Recordings were made from multiple sites along the anterior–posterior axis of A1 at a depth of 400 μm. CF, the frequency with the lowest threshold, was identified for each recording site to confirm tonotopy expected for A1, including a reversal of tonotopy where A1 borders with the anterior auditory field (Stiebler et al., 1997).

At the A1 site with shortest-latency and largest-amplitude LFPs, a 16-channel multiprobe (2–3 MΩ at 1 kHz for each 177 μm^2^ recording site, 100 μm separation between sites; NeuroNexus Technologies) was inserted perpendicular to the cortical surface. CF was re-determined using LFPs at a 300–400 μm depth. For data collection, CF stimuli were delivered at 1/s in sets of 25 trials, at a level 40 dB above threshold. NonCF stimuli, two octaves below CF, were delivered at the same intensity.

Nicotine hydrogen tartrate (Sigma) was dissolved in saline (2.1 mg/kg free base) and delivered via subcutaneous injection. Because the effects of systemic nicotine on tone-evoked responses last at least 30 min (Kawai et al., 2011; Intskirveli and Metherate, 2012), recordings began within 1 min of drug administration and all data were collected within ~20 min. Sets of acoustic stimuli were delivered three times under each condition: pre-drug, post saline, and post nicotine.

### Data analysis

AxoGraph software was used to analyze acoustic-evoked responses, with each tone-evoked response being the average response to 25 stimuli. Current-source density (CSD) profiles were created offline from LFPs, as previously (Kawai et al., 2011; Intskirveli and Metherate, 2012). The one-dimensional CSD is the second spatial derivative of the LFP laminar profile (Muller-Preuss and Mitzdorf, 1984); conventionally, a current sink reflects the location, timing, and magnitude of underlying synaptic excitation. The middle layer current sink with the shortest CF-evoked onset latency reflects monosynaptic thalamocortical input (Intskirveli et al., 2016) and was designated the “layer 4” sink. Its response onset was defined at an amplitude three standard deviations above the baseline value determined over the 100 ms preceding the tone. Initial slope (over first 5 ms) and amplitude data were normalized to pre-drug values for each condition (saline, nicotine). Group CSDs were created as previously (Intskirveli and Metherate, 2012). Briefly, for CF-evoked responses, individual CSD profiles were normalized to the largest amplitude current sink in the pre-drug condition, aligned across animals using the current sink designated as layer 4, and averaged across animals. Layers were designated using published descriptions of their relative thickness (Anderson et al., 2009; Intskirveli and Metherate, 2012). For nonCF-evoked responses, the procedure was similar except that the largest (max) current sink was assigned to layer 2/3 for averaging across animals. Interpolated group CSD (color) profiles were created in DeltaGraph (Red Rock Software).

Statistical tests were carried out using GraphPad PRISM v9.1.2. Mean values are ± SEM. Group data were analyzed for possible sex differences using two-way repeated measures ANOVA (*α* = 0.05). Since no sex differences were found, groups were collapsed across sex and data were analyzed using two-way repeated measures ANOVA for main effects (mouse genotype and drug condition) and interactions, with *post hoc* Bonferroni’s multiple comparisons test. Comparison of pre-drug current sinks between WT and KO mice used Welch’s unpaired t-test to compare absolute amplitudes (whereas in subsequent analyses, amplitudes in saline and nicotine were normalized to pre-drug values for each CSD profile). “N” values indicate number of animals. Portions of the data collected for Figure 2 were included in a dissertation (Gil, 2021).

## Results

### Distribution and *in vitro* electrophysiology of α2 nAChR-expressing neurons in auditory cortex

**Figure 1:**
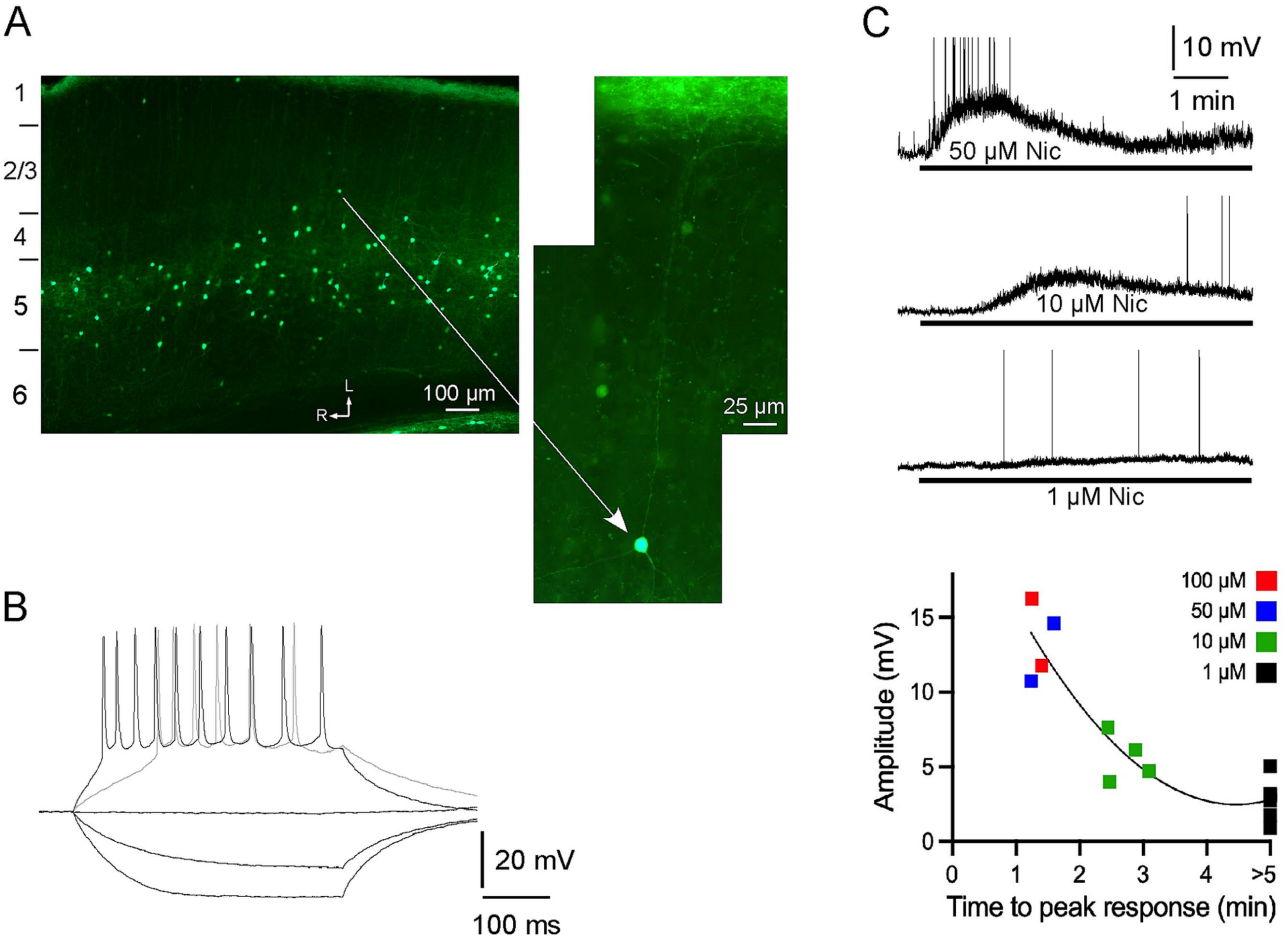
α2 nAChRs-expressing neurons in A1: distribution, intrinsic electrophysiology and response to nicotine, *in vitro*. **(A)** Neurons with EGFP are nonpyramidal, primarily in layer 5, and have processes that are largely restricted to layer 5, or extend vertically towards layer 1 (see higher power view) where they terminate in a dense band of fluorescent fibers. The imaged section is cut along the thalamocortical (near horizontal) plane and numbers on the left indicate approximate boundaries of layers (see Methods); arrows indicate lateral and rostral directions. **(B)** *In vitro* whole-cell recording showing membrane responses of a fluorescent neuron to intracellular current pulses. **(C)** *In vitro* response to bath applied nicotine (1–100 μM for >5 min) for individual neurons (top; action potentials truncated) and in group dose–response data (bottom).

We examined the cortical distribution of fluorescent label in mice expressing EGFP in cells with α2 nAChRs. The distribution of cells was entirely consistent with descriptions from an independent mouse line (Chrna2-Cre) with α2 nAChR-expressing cells labeled (Hilscher et al., 2017). A band of brightly fluorescent somata and processes was primarily in layer 5 (Figure 1A). Labeled somata were nonpyramidal and exhibited processes that were largely restricted to layer 5 or extended vertically towards layer 1 where they terminated in a dense band of fluorescent fibers. These results are consistent with previous reports that α2 nAChR-expressing neurons in cortex are layer 5 MCs with axons projecting to and arborizing extensively in superficial cortex, especially layer 1 (see Discussion).

Whole-cell recordings were obtained from fluorescent neurons in layer 5 of brain slices maintained *in vitro*. In response to hyperpolarizing current pulses the neurons exhibited little sag and input resistance of 462 ± 52 M-ohm (n = 14) (Figure 1B). Depolarizing current elicited action potentials, and stronger stimulation led to spike trains with moderate adaptation. Bath application of nicotine (1–100 μM for >5 min) produced a dose-dependent depolarization of ~1–15 mV, with higher doses producing peak depolarization within 1–3 min followed by apparent slow desensitization in the continued presence of nicotine (Figure 1C). The lowest nicotine dose (1 μM) did not produce apparent desensitization with nicotine application for at least 5 min. Thus, fluorescent neurons were sensitive to nicotine and exhibited intrinsic electrophysiology consistent with that of layer 5 MCs (Hilscher et al., 2017).

### *In vivo* CSD response to CF stimuli

**Figure 2:**
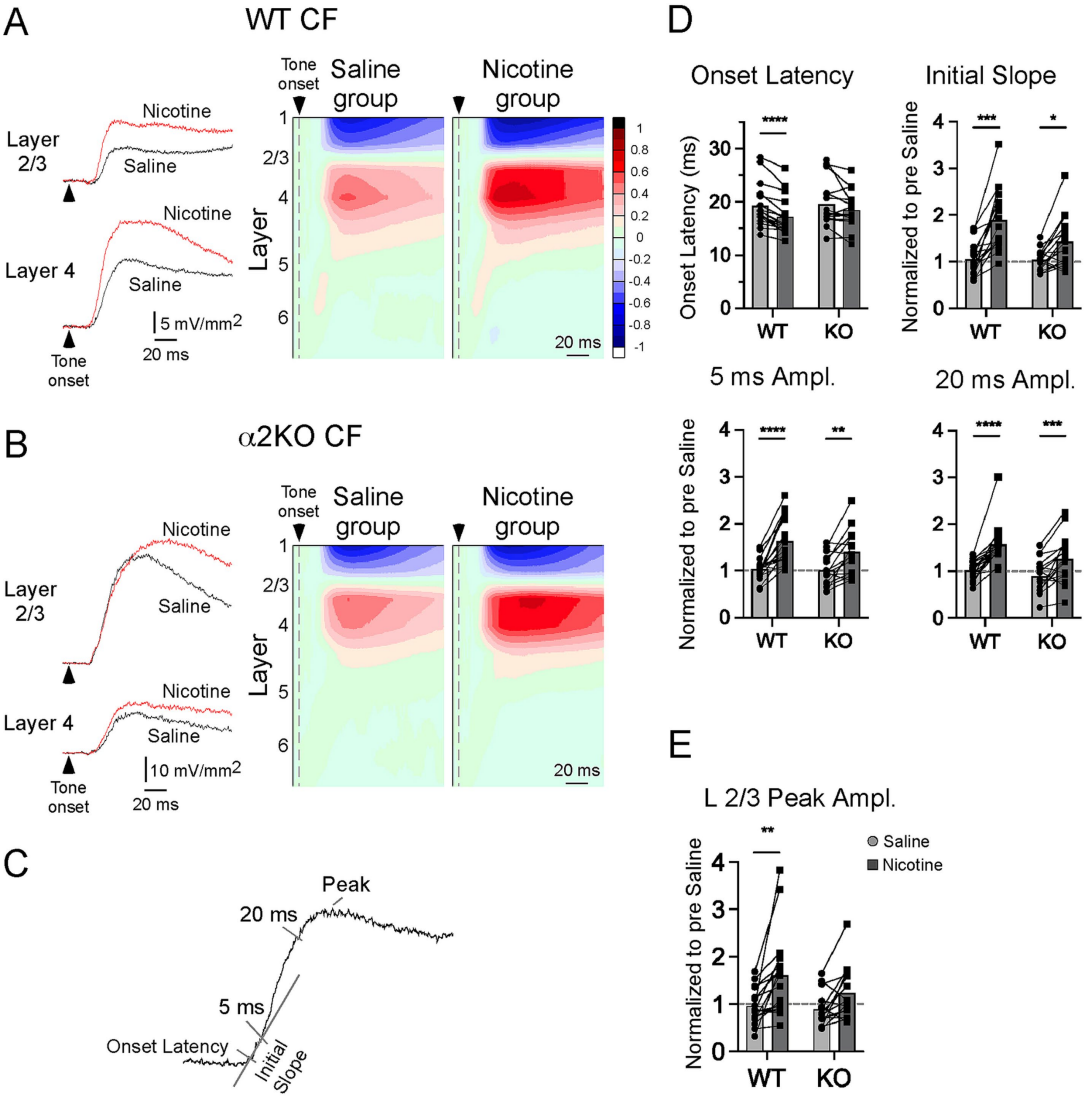
*In vivo* CSD response to CF stimuli. **(A)** In WT mice, effects of systemic saline and nicotine (2.1 mg/kg) on CF-evoked responses are shown for example current sinks (left) and group CSD profiles (right, CSDs normalized to largest pre-drug sink amplitude in each of n = 17 mice). **(B)** Effects of saline and nicotine in α2 KO mice, n = 16 mice. **(C)** Measurements to quantify effects of saline and nicotine in **(D,E)**. **(D)** Effects of saline and nicotine, with amplitude measures normalized to pre-drug values, on layer 4 current sink in WT and KO mice. **(E)** Effects of saline and nicotine on layer 2/3 current sink peak. Asterisks indicate *p* < 0.05 (*), *p* < 0.002 (**), *p* < 0.0002 (***), *p* < 0.0001 (****).

Using a 16-channel multiprobe inserted into A1, we recorded tone-evoked LFPs and derived CSD profiles in α2 KO mice and WT littermates. CSD profiles elicited by CF stimuli (40 dB above threshold) in WT mice were as described (Kawai et al., 2011; Intskirveli and Metherate, 2012), with the shortest onset-latency current sink in the middle layers designated as the “layer 4” sink and a prominent upper layer current sink as the “layer 2/3” sink (typically, one or two recording sites above the layer 4 sink; Figure 2A). The onset latency of the layer 4 current sink did not differ between WT and α2 KO mice (WT 19.53 ± 0.94 ms, KO 19.13 ± 1.04 ms; Welsh’s unpaired t-test, t = 0.28, *p* = 0.78). Similarly, the layer 4 current sink’s initial slope and amplitude at 5 ms and 20 ms (see Figure 2C) also did not differ (initial slope: WT 1.18 ± 0.18 μV/ms, KO 1.18 ± 0.29 μV/ms, t = 0.01, *p* = 0.99; amplitude at 5 ms: WT 5.67 ± 0.77 μV, KO 5.72 ± 1.40 μV, t = 0.03, *p* = 0.97; amplitude at 20 ms: WT 12.69 ± 1.51 μV, KO 14.69 ± 2.21 μV, t = 0.75, *p* = 0.46). However, for the current sink in layer 2/3, the peak amplitude was larger in KO mice (WT 12.80 ± 1.85 μV, KO 20.80 ± 3.04 μV, t = 2.24, *p* = 0.03). Thus, WT and α2 KO mice had nearly identical CF-evoked current sinks in layer 4, but KO mice had a larger current sink in layer 2/3 (*cf.* saline data in Figures 2A,B). This difference in layer 2/3 may reflect altered development of cortical circuits and/or reduced inhibition in layer 2/3 of mice lacking α2 nAChRs.

Systemic nicotine (2.1 mg/kg, s.c.) in WT mice enhanced the CF-evoked CSD profile as previously described (Kawai et al., 2011; Intskirveli and Metherate, 2012), decreasing the onset latency of the layer 4 current sink and increasing the magnitude of current sinks in layer 4 and layer 2/3 (Figure 2A, example current sinks on left, group CSD profiles on right; n = 17 mice). Saline injections delivered prior to nicotine in the same mice had no effect on pre-drug measures. Conversely, in α2 KO mice, nicotine did not affect the onset latency in layer 4 and only enhanced longer-latency portions of the current sinks (Figure 2B; n = 16). The effects of saline and nicotine were measured as in Figure 2C (normalized to pre-drug values), using onset latency, initial slope (over first 5 ms), and response amplitudes at 5 ms and 20 ms for the layer 4 sink, and peak amplitude for the layer 2/3 sink, with results shown in Figures 2D,E (two-way, repeated measures ANOVA with *post hoc* Bonferroni multiple comparisons test). For the layer 4 onset latency, relative to saline, nicotine decreased latency for WT mice (*p* < 0.00001) but had no effect in α2 KO mice (*p* = 0.2018). For initial slope, both WT (*p* < 0.0001) and α2 KO (*p* = 0.011) showed enhancement after nicotine, but with lesser enhancement in α2 KO mice (interaction, F_1,31_ = 5.911, *p* = 0.021). In both groups, nicotine increased current sink amplitude at 5 ms (WT, p < 0.0001; α2 KO, *p* = 0.0012) and 20 ms (WT, p < 0.0001; α2 KO, *p* = 0.0009). For the current sink in layer 2/3 (Figure 2E), nicotine increased peak amplitude in WT mice (*p* = 0.0005) but not α2 KO mice (*p* = 0.098). Thus, for CF-evoked current sinks, deletion of α2 nAChRs prevented the effect of nicotine on the layer 4 onset latency, but nicotine was progressively more effective on longer latency components of the layer 4 sink, partly enhancing the initial slope and fully enhancing later amplitudes. Yet, deletion of α2 nAChRs prevented enhancement of the layer 2/3 sink.

### CSD response to nonCF stimuli

NonCF stimuli (two octaves below CF, same intensity as CF) elicited CSD profiles that differed from CF-evoked responses, with the largest (“max”) current sink typically in layer 2/3 (n = 17/26 mice) and less often in layer 4 (n = 9/26). As with CF-evoked responses, the onset latency of the max current sink did not differ between WT and α2 KO mice (WT 28.08 ± 2.27 ms, KO 23.92 ± 1.32 ms; Welsh’s unpaired t-test, t = 1.59, *p* = 0.13), but its peak amplitude was larger in KO mice (WT 7.69 ± 1.34 μV, KO 17.13 ± 3.45 μV, t = 2.55, p = 0.02). Moreover, separating max current sinks in layer 2/3 from those in layer 4 showed that responses in KO mice were larger for layer 2/3 (WT 7.33 ± 1.95 μV, KO 19.38 ± 4.43, t = 2.49, *p* = 0.03) but not layer 4 (WT 8.20 ± 1.92, KO 11.52 ± 4.34, t = 0.69, *p* = 0.52). Together with the similar laminar pattern for CF-evoked responses (previous section), these results show that acoustic-evoked responses in α2 KO mice resemble WT mice in layer 4, but are larger in layer 2/3, and potentially reflect reduced inhibition in layer 2/3 of mice lacking α2 nAChRs.

**Figure 3:**
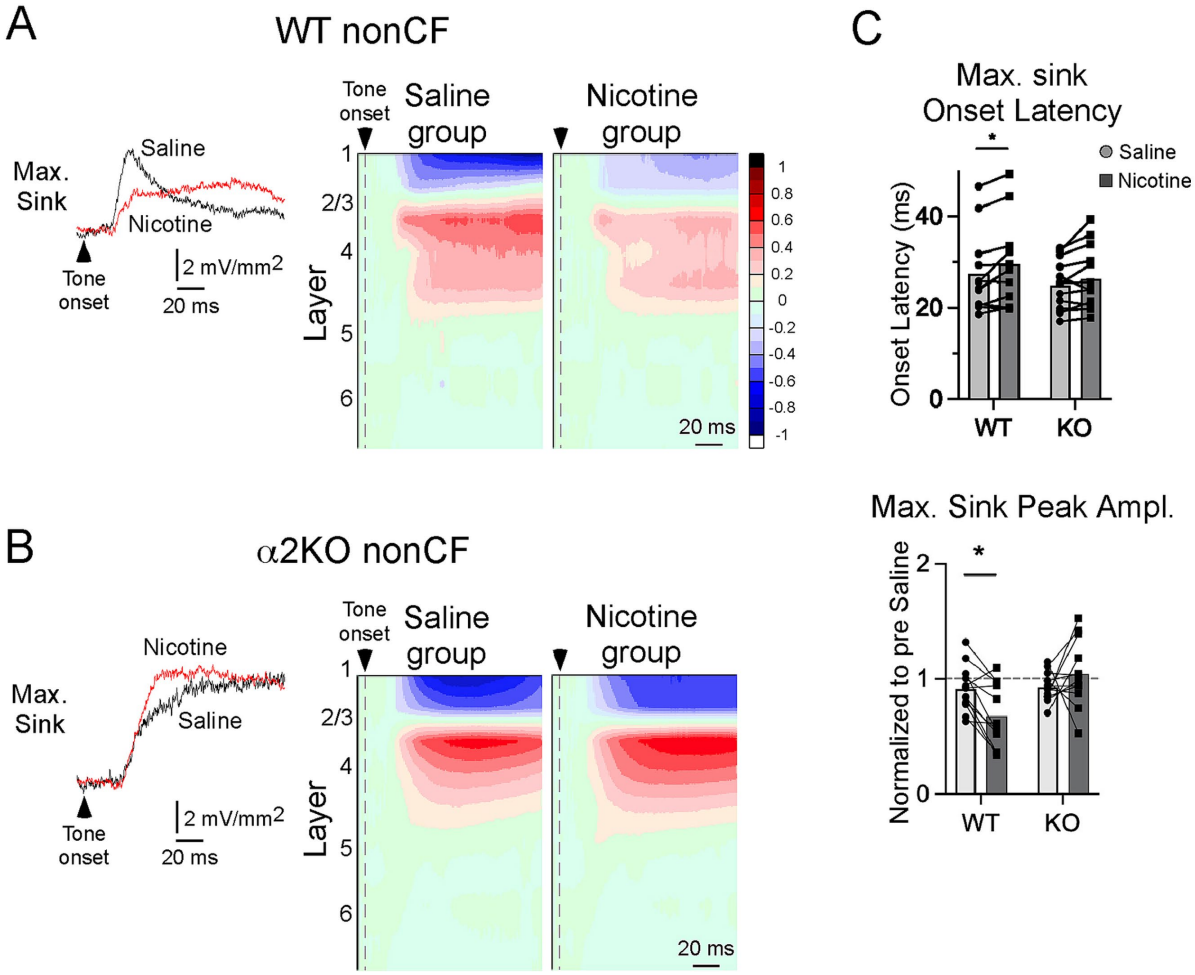
CSD response to nonCF stimuli. **(A)** In WT mice, effects of systemic saline and nicotine on the largest (max) nonCF-evoked current sink; example traces (left, from layer 2/3) and group CSD profiles (right, n = 12 mice; to illustrate the effect, max sink is aligned on L2/3 for group average). **(B)** Effects of saline and nicotine on nonCF-evoked responses in α2 KO mice, n = 13 mice. **(C)** Effects of saline and nicotine on onset latency and peak amplitude (relative to pre-drug value) of max sink in WT and KO mice. Asterisks indicate *p* < 0.02 (*).

In contrast to its effects on CF-evoked responses, systemic nicotine in WT mice suppressed the nonCF-evoked CSD profile, as described previously (Kawai et al., 2011; Intskirveli and Metherate, 2012), increasing the onset latency and decreasing the amplitude of the max sink (Figure 3A, N = 12 mice). Both effects, however, were absent in α2 KO mice (Figure 3B, N = 13). The effects of nicotine, relative to saline (delivered prior to nicotine in the same mice), on the max current sink are shown in Figure 3C (two-way, repeated measures ANOVA with *post hoc* Bonferroni multiple comparisons test). Nicotine increased onset latency in WT (*p* = 0.014) but not α2 KO mice (*p* = 0.093). Similarly, nicotine reduced the peak amplitude in WT (*p* = 0.016) but not α2 KO mice (*p* = 0.308). Thus, the effect of nicotine to suppress nonCF-evoked responses—thereby producing a narrower RF—was dependent on α2 nAChRs.

### Subcortical distribution of α2 nAChR-expressing neurons

**Figure 4:**
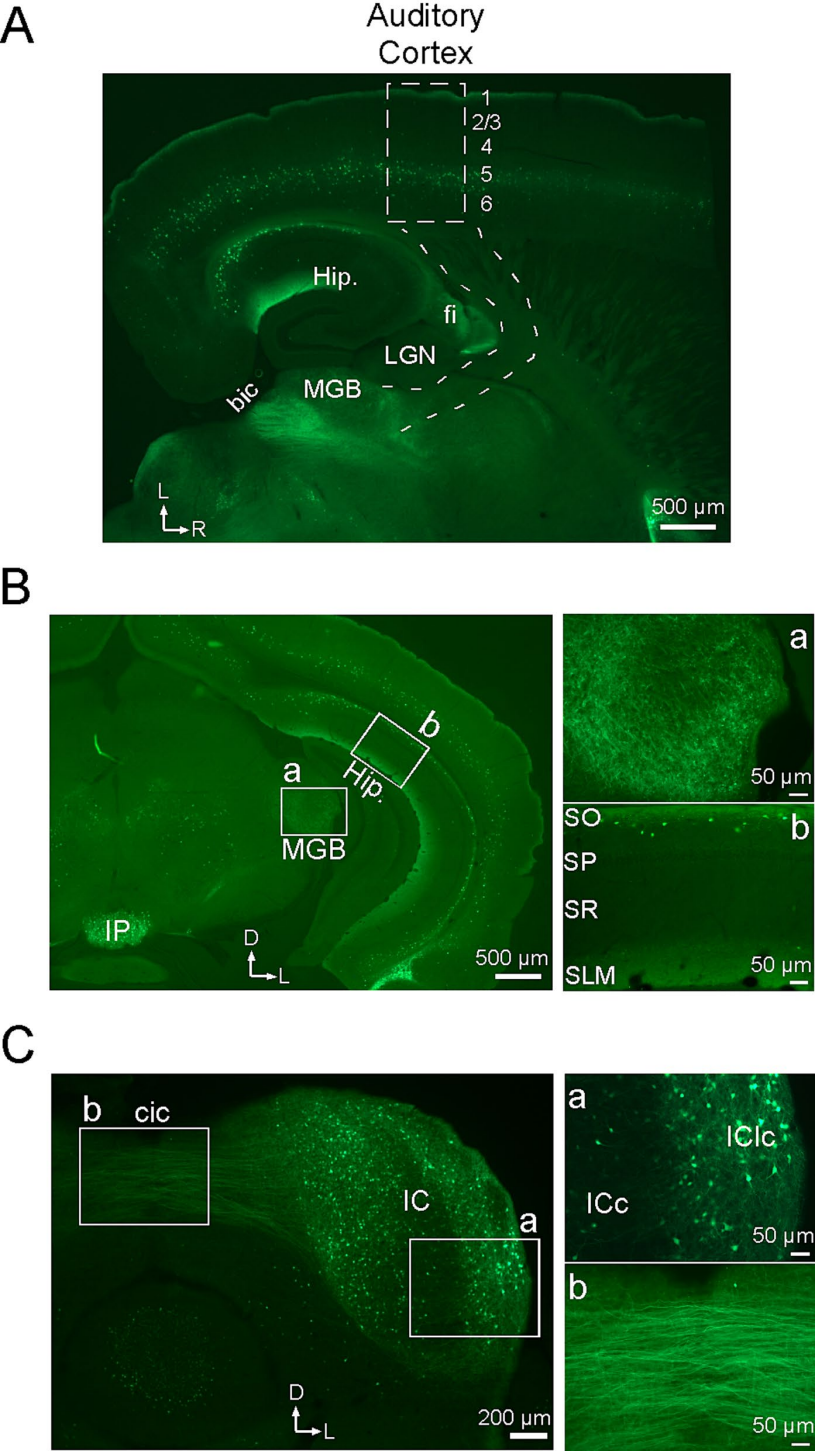
Subcortical distribution of neurons with α2 nAChRs. All sections show EGFP in cells with α2 nAChRs. **(A)** Brain section cut in the auditory thalamocortical plane (approx. 20 degrees off horizontal), showing the approximate locations of auditory cortex (dashed rectangle; numbers indicate layers) and thalamocortical pathway (dashed lines). Hip, hippocampus; LGN, lateral geniculate nucleus; MGB, medial geniculate body; fi, fimbria; bic, brachium of the inferior colliculus. **(B)** Coronal section at the level of the MGB with white rectangles showing regions of higher power views for MGB (a) and hippocampus (b). IP, interpeduncular nucleus; SO, stratum oriens; SP, stratum pyramidale; SR, stratum radiatum; SLM, stratum lacunosum moleculare. **(C)** Coronal section through the IC with higher power views of the IC (a) and intercollicular commissural pathway (b). cic, intercollicular commissure; ICc, central nucleus of the IC; IClc, lateral cortex of the IC. In all low power images, arrows indicate lateral (L), rostral (R) and dorsal (D) directions.

Since deletion of α2 nAChRs prevented systemic nicotine from reducing the onset latency of CF-evoked responses in layer 4 (which reflect thalamocortical inputs) (Figure 2), we inferred that subcortical α2 nAChRs normally play a role in regulating afferent input. We therefore examined the distribution of α2 nAChR-expressing cells in the subcortical auditory pathway. Figure 4A shows a low-power image of a brain section cut to preserve the auditory thalamocortical pathway, as per Cruikshank et al. (2002). In cortex, fluorescent cells in layer 5 and a band of label in layer 1 are seen throughout the anterior–posterior extent of the section, including auditory cortex (approximate region of rectangle in Figure 4A). However, the region of the thalamocortical pathway (subcortical dashed lines) contained no fluorescent label even when examined under high power, except within the thalamus itself (see below). Note the clear label in hippocampus where presumed oriens lacunosum-moleculare (OLM) neurons can be seen with somata in stratum oriens (SO) and dense processes corresponding to axonal arborizations in stratum lacunosum moleculare (SLM) (Figure 4A, *cf.* Figure 4B). Fluorescent fibers are also seen in the fimbria (fi), the source of cholinergic input to hippocampus, and posterior to the MGB in the brachium of the inferior colliculus (bic).

The distribution of EGFP in the auditory thalamus and midbrain is more clearly viewed in coronal sections (Figures 4B,C). Figure 4B shows a low-power section at the level of the medial geniculate body (MGB, white rectangle), with a higher power view (Figure 4Ba) showing labeled processes but no somata in the MGB. Note in the low power section that the distribution of presumed layer 5 MCs extends throughout the dorso-ventral extent of cortex, similarly to their rostro-caudal distribution in Figure 4A, and the distribution of OLM cells (at higher power in Figure 4Bb), as described in Figure 4A, extends throughout dorso-ventral extent of hippocampus. Note also the dense fluorescence near the ventral midline in the interpeduncular nucleus (IP), which reportedly exhibits the highest density of α2 nAChRs in the brain (Wada et al., 1989; Ishii et al., 2005; Whiteaker et al., 2009). Finally, the coronal section in Figure 4C shows the inferior colliculus (IC), with labeled somata and processes in the IC itself (Figure 4Ca) and prominent fibers extending into the commissural pathway (cic, Figure 4Cb). Within the IC, fluorescent label appears heaviest in non-lemniscal regions (dorsal and lateral cortex) and lighter in the central nucleus of the IC (Figure 4Ca). Coronal sections between the level of the MGB and the IC (not shown) also reveal labeled fibers in the brachium of the IC, as in Figure 4A, indicating expression of α2 nAChRs in neurons that project from IC to MGB. Thus, α2 nAChRs may be well positioned in both the MGB and IC to regulate afferent processing in subcortical auditory regions.

## Discussion

**Figure 5:**
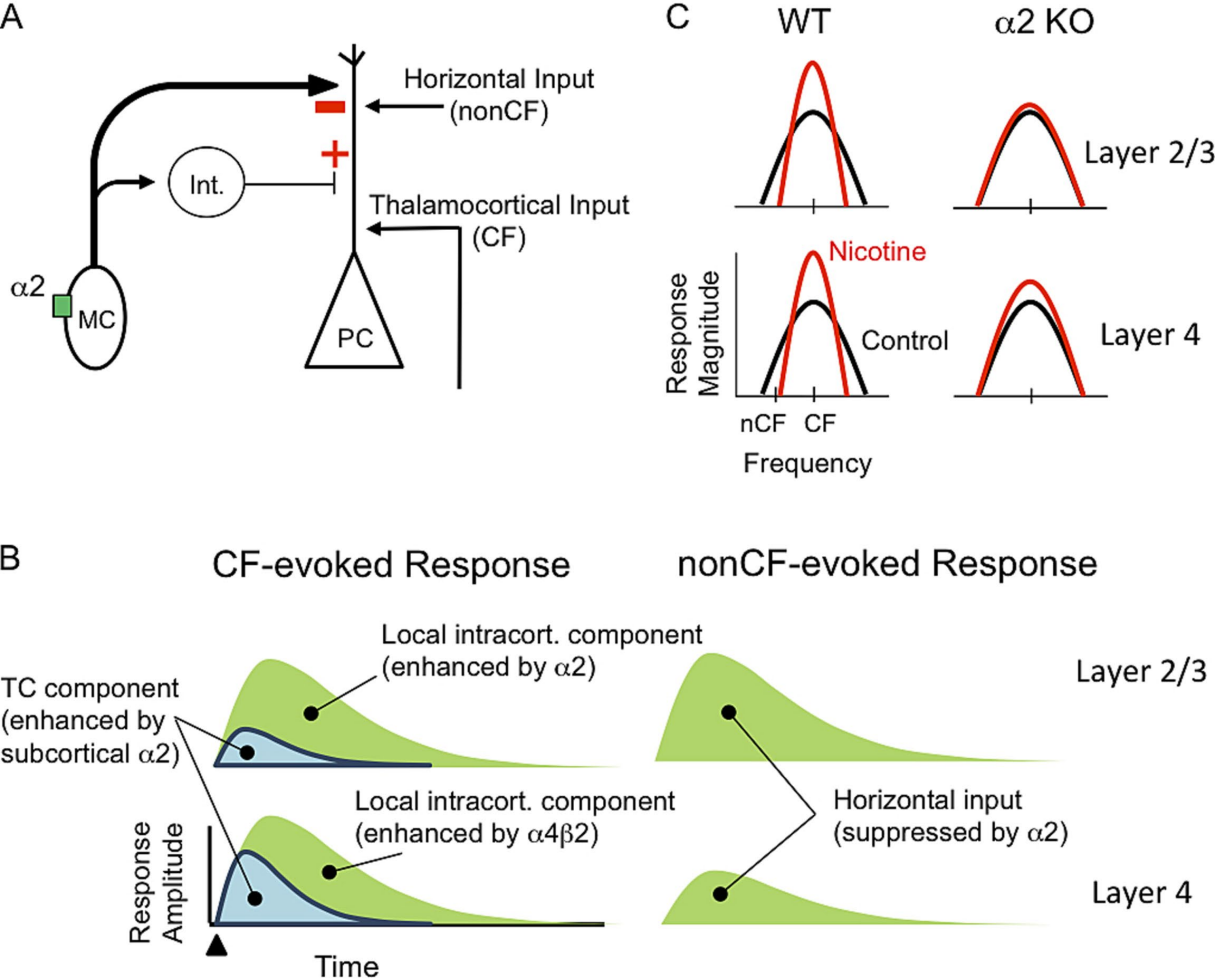
Hypothesized neural circuit and functions of α2 nAChRs. **(A)** Neural circuit with α2 nAChR in MC that projects to PC distal apical dendrite and interneuron (Int.) that, in turn, projects to PC. Nicotinic activation of MC will inhibit PC distal dendrite and disinhibit proximal dendrite. PC receives excitatory input from thalamocortical projections relaying CF information and horizontal projections relaying nonCF information. **(B)** Schematic current sinks (synaptic response) in layers 2/3 and 4 evoked by CF and nonCF stimuli. CF-evoked current sinks contain thalamocortical (TC) and local intracortical components, whereas nonCF-evoked sinks are entirely intracortical, mediated by horizontal inputs. Current sink components are differentially regulated by α2 and non-α2 (likely α4β2) nAChRs. **(C)** Hypothesized effect of systemic nicotine on RFs in layers 2/3 and 4 in WT and α2 KO mice. In WT mice nicotine sharpens RFs, i.e., increases gain and narrows RFs, in both layers. In KO mice, nicotine does not narrow RFs in either layer and only partly enhances gain in layer 4.

We tested the hypothesis that activation of α2 nAChRs—likely in layer 5 MCs—inhibits intracortical (horizontal) inputs relaying nonCF information and enhances, via disinhibition, thalamocortical inputs relaying CF information (Figure 5A). In α2 KO mice, systemic nicotine enhanced some, but not all, aspects of CF-evoked current sinks; unlike in WT mice, nicotine failed to reduce the onset latency of the thalamocortically mediated sink in layer 4, implicating subcortical α2 nAChRs—e.g., in the MGB and IC—in regulation of afferent input (Figure 5B, left). Local intracortical components of CF-evoked current sinks in layer 4 remained enhanced by nicotine in α2 KO mice, indicating contributions of non-α2 nAChRs. In layer 2/3 of α2 KO mice, nicotine did not enhance CF-evoked responses, consistent with a role for α2-MCs to disinhibit responses in layer 2/3. Of primary interest to this study, in α2 KO mice nicotine did not affect nonCF-evoked responses, indicating that α2 nAChRs normally function to suppress nonCF-evoked responses (Figure 5B, right). Inhibition of horizontal inputs could underlie suppression of nonCF-evoked responses and, therefore, RF narrowing. Together, these results support the proposed circuit (Figure 5A) whereby activation of α2 nAChRs could sharpen RFs, i.e., produce a narrowed RF and contribute to increased gain within the narrowed RF (Figure 5C).

### α2 nAChRs in auditory cortex and layer 5 Martinotti cells

Using mice that express EGFP in cells with α2 nAChRs, we observed fluorescent label in nonpyramidal somata and processes within layer 5 across many cortical regions, including auditory cortex, with thin processes ascending to a dense band of label in layer 1 (Figures 1, 4). These observations are entirely consistent with results from an independent mouse line (Hilscher et al., 2017). That study also individually filled and reconstructed labeled cells to demonstrate that all are layer 5 MCs with axon projections locally near the soma and a characteristic main axon projecting to layer 1. MCs expressing α2 nAChRs were found only in layer 5 and were a subset of MCs and other SOM-expressing interneurons in multiple cortical layers. These results also are consistent with *in situ* hybridization studies showing α2 nAChR mRNA in infragranular interneurons throughout cortex (and in hippocampal OLM cells) (Ishii et al., 2005). As in the prior studies, in the present dataset α2 nAChR-expressing neurons in layer 5 often express SOM and are a subset of SOM interneurons seen in multiple layers (unpublished observations). Notably, the present study showed that labeled cells (presumed layer 5 MCs) respond to nicotine with a small amplitude depolarization (Figure 1C), similar to the response of MCs to optogenetic stimulation of cholinergic afferents (Obermayer et al., 2018).

Generally, MCs are bipolar interneurons that often express SOM, can be found in any layer, and have axons with spiny boutons that ascend towards layer 1 (Wang et al., 2004). MCs in layer 5, the original MC described by Martinotti and Cajal, are defined by a prominent axon projection to layer 1 with an extensive terminal arborization that spreads horizontally across multiple cortical columns (Hilscher et al., 2023). Studies have identified two MC subtypes based on the pattern of terminal arborization: the terminals of T-shaped MCs arborize extensively within layer 1, whereas fanning-out MCs include terminal arborizations in layer 2/3 (Nigro et al., 2018). Both types of MC have axonal and dendritic processes near their soma in layer 5. Our hypothesized regulation of horizontal inputs that terminate in multiple superficial layers, not only layer 1, suggests that fanning-out MCs regulate nonCF inputs.

In response to intracellular current pulses, layer 5 MCs exhibit spike-frequency adaptation (Hilscher et al., 2017) as in our study (Figure 1B). T-shaped MCs also exhibit low-threshold spikes (Nigro et al., 2018), which were not observed in our small sample. Dual-cell recordings show that MCs inhibit PCs, with dendritic recordings indicating that the inhibition originates in PC apical dendrites, not the soma, and electron microscopy revealing multiple synaptic contacts along the length of distal apical dendrites (Silberberg and Markram, 2007). Dual-cell recordings also reveal less common MC projections to supragranular interneurons that, in turn, inhibit PCs near the soma (Donato et al., 2023). Thus, layer 5 MCs could mediate bidirectional regulation of PCs: inhibition of distal apical dendrites and disinhibition of proximal dendrites and/or the soma (Figure 5A). A similar neural circuit in the hippocampal CA1 region involves OLM interneurons that also express α2 nAChRs and SOM, and can inhibit or disinhibit spatially distinct inputs to PC distal or proximal apical dendrites, respectively (see Introduction) (Nakauchi et al., 2007; Leao et al., 2012; Hilscher et al., 2023). The location of OLM somata in SO with prominent axonal arborization in SLM is consistent with the observed distribution of labeled somata and processes in the present study (Figures 4A,B).

### Acoustic-evoked responses in A1: a functional role for α2 nAChRs

In WT mice, systemic nicotine enhances CF-evoked current sinks in layer 4 and layer 2/3, decreasing the onset latency in layer 4 and increasing the magnitude of both sinks (Figure 2) (Kawai et al., 2011; Intskirveli and Metherate, 2012). CF inputs are carried by thalamocortical projections that contact neurons in the middle layers, including proximal apical dendrites of PCs, to produce the layer 4 current sink. Pharmacological silencing of intracortical activity to isolate CF-evoked thalamocortical inputs has shown that the initial portion of the layer 4 sink reflects monosynaptic thalamocortical input, whereas longer latency components reflect progressively greater contributions from local intracortical sources (Figure 5B) (Intskirveli et al., 2016). Thus, the present results from α2 KO mice showing that systemic nicotine had no effect on onset latency, partially enhanced the initial slope, and fully enhanced longer-latency components suggest that deletion of α2 nAChRs eliminates nicotinic enhancement of thalamocortical inputs but does not prevent enhancement of local intracortical activity via, presumably, α4β2 nAChRs (Figure 5B) (Kawai et al., 2011; Intskirveli and Metherate, 2012). These results also point to a subcortical role in WT mice for α2 nAChRs to mediate some effects of systemic nicotine (see below).

The CF-evoked current sink in layer 2/3 involves proportionally greater intracortical activity (compared to layer 4) generated by local cortical circuits (Intskirveli et al., 2016). The layer 2/3 sink is also enhanced by systemic nicotine, an effect that is absent in α2 KO mice (Figure 2E), thereby implicating a role for α2 nAChRs to enhance local intracortical activity activated by CF stimuli (Figure 5B).

However, nonCF stimuli (two octaves below CF) typically elicit a large current sink in layer 2/3 (Figure 3A) that is fully eliminated by pharmacological silencing of intracortical activity (Intskirveli et al., 2016). This result indicates that nonCF-evoked responses are due to intracortical horizontal projections that arise in distant A1 regions where the stimulus is CF (Happel et al., 2010; Kratz and Manis, 2015; Intskirveli et al., 2016). Systemic nicotine in WT mice suppresses nonCF-evoked current sinks to narrow the breadth of RFs (Figure 3). This effect is mimicked by intracortical injection of a positive allosteric modulator of α2 and α4 nAChRs (Askew et al., 2017) and blocked by intracortical injection of a drug to inhibit nicotinic activation of MAPK (Intskirveli and Metherate, 2012), indicating a cortical locus, at least in part, for RF narrowing resulting from either nicotine or endogenous ACh (note that systemic nicotine also narrows RFs in the IC but this result cannot fully explain regulation of the significantly broader RFs in cortex (Askew et al., 2017)). Notably, in *α*2 KO mice, nicotine has no effect on the nonCF-evoked sink (Figure 3), implicating α2 nAChRs in the suppression of horizontal inputs (Figure 5B). Moreover, although we did not assess the full breadth of RFs in this study, the larger pre-drug amplitude of nonCF-evoked sinks in α2 KO mice, relative to WT mice, suggests that KO mice have broader RFs, possibly reflecting reduced inhibition of horizontal projections that relay nonCF inputs. Such reduced inhibition could reflect a reduced effect of endogenous ACh release on layer 5 MCs (Obermayer et al., 2018) due to the absence of α2 nAChRs, or related effects resulting from altered development in KO mice.

Thus, the opposite effects of systemic nicotine on CF- vs. nonCF-evoked responses in WT mice, and the differential effects on these responses produced by deletion of α2 nAChRs, suggest differential regulation by α2 nAChRs of thalamocortical inputs, local intracortical activity, and long-distance horizontal inputs (Figure 5B). The results are consistent with a role for layer 5 MCs expressing α2 nAChRs to differentially regulate thalamocortical and horizontal inputs to PCs (Figure 5A). We hypothesize that through this mechanism, α2 nAChRs contribute to RF sharpening by suppressing responses to nonCF stimuli and contributing (with α4*β*2 nAChRs) to enhanced CF-evoked responses (Figure 5C).

### Subcortical α2 nAChRs

In α2 KO mice, the absence of nicotine’s effect on the onset latency of the CF-evoked current sink suggests that subcortical α2 nAChRs normally act to enhance thalamocortical inputs. While the locus of action is unknown, one possibility is the thalamocortical pathway where anatomical binding studies indicate the presence of heteromeric nAChRs in rodents and primates (Ding et al., 2004; Bieszczad et al., 2012; Mukherjee et al., 2017), and physiological studies demonstrate nicotinic actions to increase the excitability of myelinated thalamocortical axons (Kawai et al., 2007). However, the present study did not reveal clear anatomical evidence for α2 nAChRs in the thalamocortical pathway, i.e., there was little fluorescent label in the pathway and none in somata of MGB neurons (Figures 4A,B). This absence of label is puzzling given: (i) evidence that nAChRs located in the thalamocortical pathway regulate input to cortex (Kawai et al., 2011; Intskirveli and Metherate, 2012; Askew et al., 2017), (ii) evidence that nAChRs in the pathway contain β2 subunits (e.g., α4β2 or α2β2) (Kawai et al., 2011; Bieszczad et al., 2012; Intskirveli and Metherate, 2012; Askew et al., 2017), and (iii) in α2 KO mice, the lack of nicotine effect on the layer 4 input, rejecting a role for α4 nAChRs and implicating α2 nAChRs (present study). That is, physiological results implicate α2 nAChRs in the thalamocortical pathway, but anatomical evidence is lacking. It may be that α2 nAChRs are spatially segregated to regulate nodes of Ranvier on thalamocortical axons, or otherwise have low levels of expression, and are not readily visible in the present study (e.g., note the weak fluorescence where thalamocortical axons exit the MGB; Figure 4A). Closer examination of nAChR subunits in the thalamocortical pathway may resolve this issue.

Fluorescent label for α2 nAChR-expressing cells was also seen in subcortical auditory relay nuclei, including labeled processes in the MGB and both processes and somata in the IC (Figures 4B,C). The source of labeled processes in the MGB is unknown, but the presence of labeled fibers in the brachium of the IC suggests that α2 nAChRs are found in IC neurons that project to the MGB. Studies of MGB neurons have shown nicotinic presynaptic regulation of inhibitory, but not excitatory, inputs from IC (Sottile et al., 2017). In addition, inhibitory neurons in the IC contain mRNA for β2 nAChRs but not α4 nAChRs (Sottile et al., 2017). Since heteromeric nAChRs contain both α and β subunits, these results together suggest the possibility of α2β2 nAChRs on the terminals of IC inhibitory projections to MGB. Finally, fiber label in the midbrain was prominent in the commissural pathway to the contralateral IC (Figure 4C). The IC receives extensive cholinergic input (Beebe and Schofield, 2021; Noftz et al., 2024) and nAChRs mediate postsynaptic excitation of IC neurons (Rivera-Perez et al., 2021). Notably, the projection pattern to the IC from one brainstem cholinergic nucleus, the lateral paragigantocellular nucleus, resembles the distribution of fluorescent label in the present study (Figure 4C), generally avoiding the central subdivision of the IC and projecting more heavily to surrounding, non-lemniscal regions (Noftz et al., 2024). Thus, while α2 nAChRs may contribute to both presynaptic and postsynaptic nicotinic actions in subcortical auditory nuclei, the contribution of these actions to cortical RF sharpening remains to be determined.

### Sustained activation of α2 nAChRs and broader implications

The neuromodulatory effects of systemic nicotine on acoustic responses endure for tens of minutes after a single subcutaneous injection (Kawai et al., 2011; Intskirveli and Metherate, 2012). Such long-lasting effects may involve sustained activation of α2 nAChRs that exhibit less desensitization in the continued presence of agonist, compared to other nAChRs (Sudweeks and Yakel, 2000). In hippocampal OLM cells, α2 nAChRs do not desensitize during 1 μM nicotine application for many minutes (Jia et al., 2009). In the present study we observed sustained depolarization *in vitro* at a similar dose (1 μm for 5 min, Figure 1C), but apparent slow desensitization at higher doses. However, the apparent desensitization could instead reflect recruitment of inhibitory process; further studies with pharmacologically isolated cells will be needed to resolve this issue. In addition, prolonged effects of systemic nicotine on auditory processing have been shown to involve MAPK signal transduction triggered by activation of nAChRs in A1 (Intskirveli and Metherate, 2012). Regardless of the mechanism, sustained activation of α2 nAChRs by systemic nicotine, or tonic release of endogenous ACh, could produce lasting modulatory effects to sharpen RFs in A1, potentially mimicking (for nicotine) or producing (for ACh) the RF sharpening that occurs during auditory attention (Okamoto et al., 2007; Lakatos et al., 2013; O'Connell et al., 2014). To exploit this effect therapeutically, the effects of nicotine on human psychoacoustics is being investigated as a potential treatment for auditory-attention disorders (Pham et al., 2020; Sun et al., 2021).

Nicotinic activation of MCs undoubtedly exerts effects beyond the auditory modality. MCs could inhibit long distance inputs to A1, such as descending inputs from frontal cortex (Zhang et al., 2014; Ledderose et al., 2023), potentially changing the nature of cortical processing to favor sensory inputs over top-down regulation. Similar ideas have been proposed for hippocampus where activation of OLM cells may switch the mode of information processing (Dannenberg et al., 2017; Hilscher et al., 2023). The similar distribution of α2 nAChRs throughout much of cortex and hippocampus (Figures 4A,B) suggests a widespread role for α2 nAChRs to mediate effects that are similar at a local circuit level (Hilscher et al., 2023), but with widely varying functional consequences across different cortical and hippocampal regions. The integrated effects could contribute to the cognitive-enhancing effects of systemic nicotine (Warburton, 1992; Levin et al., 2006; Sarter et al., 2009; Newhouse et al., 2012; Gil and Metherate, 2019).

## Data Availability

The original contributions presented in the study are included in the article/supplementary material, further inquiries can be directed to the corresponding author.
